# Developmental myosins: expression patterns and functional significance

**DOI:** 10.1186/s13395-015-0046-6

**Published:** 2015-07-15

**Authors:** Stefano Schiaffino, Alberto C. Rossi, Vika Smerdu, Leslie A. Leinwand, Carlo Reggiani

**Affiliations:** Venetian Institute of Molecular Medicine (VIMM), Via G. Orus 2, 35129 Padova, Italy; Department of Molecular, Cellular and Developmental Biology and BioFrontiers Institute, University of Colorado, Boulder, CO USA; Institute of Anatomy, Faculty of Medicine, University of Ljubljana, Ljubljana, Slovenia; Department of Biomedical Sciences, University of Padova, Padova, Italy; CNR Institute of Neuroscience, Padova, Italy

**Keywords:** Muscle development, Muscle regeneration, Myosin heavy chain, Embryonic myosin, Neonatal myosin, Distal arthrogryposis

## Abstract

Developing skeletal muscles express unique myosin isoforms, including embryonic and neonatal myosin heavy chains, coded by the myosin heavy chain 3 (*MYH3)* and *MYH8* genes, respectively, and myosin light chain 1 embryonic/atrial, encoded by the myosin light chain 4 (*MYL4)* gene. These myosin isoforms are transiently expressed during embryonic and fetal development and disappear shortly after birth when adult fast and slow myosins become prevalent. However, developmental myosins persist throughout adult stages in specialized muscles, such as the extraocular and jaw-closing muscles, and in the intrafusal fibers of the muscle spindles. These myosins are re-expressed during muscle regeneration and provide a specific marker of regenerating fibers in the pathologic skeletal muscle. Mutations in *MYH3* or *MYH8* are responsible for distal arthrogryposis syndromes, characterized by congenital joint contractures and orofacial dysmorphisms, supporting the importance of muscle contractile activity and body movements in joint development and in shaping the form of the face during fetal development. The biochemical and biophysical properties of developmental myosins have only partially been defined, and their functional significance is not yet clear. One possibility is that these myosins are specialized in contracting against low loads, and thus, they may be adapted to the prenatal environment, when fetal muscles contract against a very low load compared to postnatal muscles.

## Review

### Introduction

Sarcomeric myosins present in mammalian striated muscle are class II or conventional myosins, each myosin molecule consisting of two heavy chains (MyHCs), two essential light chains (MLCs), and two regulatory MLCs. Both MyHCs and MLCs are present in different isoforms encoded by different genes. A total of 11 MyHCs is coded by 6 myosin heavy chain (*MYH*) genes which are widely expressed in body muscles and 5 other genes with limited expression in specialized skeletal muscles. Five essential MLCs are coded by four myosin light chain (*MYL*) genes, and two regulatory MLCs by two other *MYL* genes (Table 1) (see [1]). Most of these genes are also expressed in the developing skeletal muscle, including two MyHC isoforms, called embryonic and neonatal (or perinatal) myosins, coded by *MYH3* and *MYH8*, respectively, and myosin light chain 1 embryonic/atrial, coded by the *MYL4* gene, which are present at high levels in the initial stages of muscle development, are downregulated after birth, and are re-expressed during muscle regeneration. Here, we review the pattern of expression of myosin genes during muscle development, focusing especially on embryonic and neonatal MyHCs. In addition, we discuss the human pathologies due to mutation of *MYH3* and *MYH8* and the unsettled question of the functional significance of these myosins.

**Table 1:** *MYH* and *MYL* genes expressed in developing mammalian skeletal muscle

Protein	Gene	Expression in developing muscle	Expression in adult muscle
Myosin heavy chains^a^			
MyHC-emb	*MYH3*	Embryonic and fetal muscle	Specialized muscles^b^
MyHC-neo	*MYH8*	Embryonic and fetal muscle	Specialized muscles^b^
MyHC-slow	*MYH7*	Embryonic and fetal muscle	Type 1 muscle fibers and ventricles
MyHC-2A	*MYH2*	Fetal (human) or early postnatal (mouse) muscle	Type 2A muscle fibers
MyHC-2X	*MYH1*	Late fetal (human) or early postnatal (mouse) muscle	Type 2X muscle fibers
MyHC-2B	*MYH4*	Postnatal muscle	Type 2B muscle fibers
Essential myosin light chains^c^			
MLC-1fast^d^	*MYL1*	Embryonic muscle	Fast muscle
MLC-3fast^d^	*MYL1*	Fetal muscle (mouse: after E15)	Fast muscle (2B > 2A)
MLC-1emb/atrial	*MYL4*	Embryonic muscle, heart	Atria
MLC-1sb	*MYL3*	Fetal muscle (mouse: after E15)	Slow skeletal muscle and ventricles
MLC1-sa	*MYL6B*	Fetal muscle (human)	Slow skeletal muscle, not ventricles, in human, not mouse
Regulatory myosin light chains			
MLC-2fast	*MYLPF*	Embryonic and fetal muscle	Fast muscle
MLC-2slow	*MYL2*	Embryonic and fetal muscle	Slow muscle and ventricles

^a^Other five MyHCs coded by genes with limited expression in specialized skeletal muscles (*MYH6*, *MYH7b*, *MYH13*, *MYH15*, and *MYH16*) are not considered in this Table (see [1])

^b^Extraocular, masticatory, laryngeal muscles, and muscle spindles

^c^An additional MLC, coded by the *MYL6* gene, which is normally expressed in smooth muscle and non-muscle cells, is detectable in human fetal muscle and human cultured muscle cells [50]. However, it is not clear whether this MLC is associated to sarcomeric myosins

^d^Splicing product of the *MYL1* gene

### Identification of developmental myosins in mammalian skeletal muscle

A number of studies in the 1960s and 1970s reported biochemical evidence suggesting that myosins isolated from mammalian embryonic or fetal skeletal muscle differ from adult muscle myosins (see references in [2, 3]). However, Whalen et al. [2] were the first to provide unambiguous evidence for the existence of distinct developmental myosins. They identified two specific MyHCs, called embryonic and neonatal (also called perinatal) MyHCs, hereafter referred to as MyHC-emb and MyHC-neo, which precede the appearance of adult fast myosins in the developing rat skeletal muscle [2]. The corresponding *MYH* genes were identified [4, 5] and found to be located in the same chromosomal locus as gene coding for adult fast myosin heavy chains on chromosome 11 (mouse) or 17 (human) [6]. The gene coding for MyHC-neo (*MYH8*) shows considerable sequence similarity with adult fast *MYH* genes, whereas the gene coding for MyHC-emb (*MYH3*) is quite different (see [7] for a comparative sequence analysis of *MYH* genes). Embryonic skeletal muscles also contain a unique type of essential MLC, MLC-1emb, encoded by the *MYL4* gene, which is also expressed in the developing heart and in adult atrial myocardium but not in adult skeletal muscle [8, 9].

### Developmental myosins in other vertebrates

Developmental myosins are also present in other vertebrates, such as birds and fish, although the sarcomeric myosin gene families are still incompletely characterized in these species. The identification of developmental myosins in fish is complicated by the large number of myosin genes, resulting from whole-genome duplication [10]. In the zebrafish embryo, diversification of fast and slow muscle cell lineages occurs very early in development, under the control of specific signaling pathways, leading to regional specification of different fast and slow MyHC isoforms. Three slow-type myosin genes, *smyhc1*, *smyhc2*, and *smyhc3*, that form a tandem array in the genome, show differential expression patterns, with primary slow fibers predominantly expressing *smyhc1* and secondary slow fibers, which are formed later in development, expressing *smyhc2* and *smyhc3* [11]. Six fast-type myosin genes, arranged as triple repeats located in a narrow region on opposite strands of chromosome 5, also display distinct expression patterns in the zebrafish embryo: the genes in group 1 (*fmyhc1.1*, *fmyhc1.2*, and *fmyhc1.3*) are excluded from the tail and the majority of the cranial muscle, whereas the genes in group 2 (*fmyhc2.1*, *fmyhc2.2*, and *fmyhc2.3*) are highly expressed in the cranial muscles [12].

In birds, three embryonic and one neonatal MyHC have been identified in developing skeletal muscles (reviewed by [13]). In addition, the myotome and the developing muscles in chick embryo contain three slow-type MyHCs, referred to as SM1 (or MyHC1), SM2 (or MyHC2), and SM3 (or MyHC3), SM3 being also expressed in the atrial myocardium [13, 14]. Ventricular MyHC is also transiently expressed in the embryonic chick skeletal muscles and is re-expressed during muscle regeneration [14]. The expression of slow-type MyHCs occurs in specific skeletal muscles independently of innervation, as a result of the existence of distinct lineages of myogenic precursors (see [15]). The switching from developmental to adult isoforms also varies in different chicken muscles: a complete switch from embryonic/neonatal-to-adult fast MyHC occurs in the pectoralis muscle, but most other muscles contain embryonic/neonatal isoforms as major components throughout adult stages [13].

### Embryonic and neonatal myosins during rat and mouse muscle development

Embryonic and neonatal myosins have been especially well characterized in developing rat and mouse skeletal muscles. MyHC-emb and MyHC-neo transcripts have been detected by in situ hybridization in the early developmental stages: in the mouse embryo, MyHC-emb is first detected at 9.5 days post coitum (E9.5) and MyHC-neo at E10.5 [16]. The upregulation of these genes is apparently controlled by the activity of the myogenic regulatory factors MyoD and Myf5, involved in muscle commitment and differentiation, as the proximal promoters of developmental myosin genes contain E-boxes responding to MyoD and Myf5 [17, 18]. The developing skeletal muscles also express a myosin indistinguishable from the adult MyHC-slow, coded by *MYH7*, as determined by analyses at the protein and transcript level [19]. Based on the pattern of reactivity of a number of anti-myosin antibodies, it was suggested that the slow-type MyHC isoforms present in the embryonic muscles are actually different from those present in the adult skeletal muscle [20]; however, this interpretation has not been confirmed. It was also suggested that the slow-tonic MyHC, first identified in the extraocular muscles and intrafusal fibers of muscle spindles of the adult muscles [21] and recently found to be coded by the *MYH7b* gene [22], is a slow-developmental isoform widely expressed in most embryonic muscles [23]. However, *MYH7b* transcripts are present at very low levels in embryonic mouse muscle at E12, and MYH7b protein is not detected in embryonic and fetal muscle using a polyclonal antibody specific for the N-terminal domain of MYH7b, except for rare fibers, first identified around E20, destined to become the bag fibers of muscle spindles (see [22]). In conclusion, available evidence indicates that three MyHCs are present at the protein level in the developing rat and mouse skeletal muscle: MyHC-emb (*MYH3*), MyHC-neo (*MYH8*), and MyHC-slow (*MYH7*). Immunohistochemical studies showed that the pattern of expression of developmental myosins varies in fibers formed at different developmental stages. In rat primary generation fibers, MyHC-emb is co-expressed with MyHC-slow [19, 24], whereas secondary generation fibers express embryonic and neonatal MyHCs [25]. At the later fetal stages, a number of primary generation fibers tend to lose MyHC-slow and acquire MyHC-neo reactivity, while a number of secondary generation fibers in slow muscles stain also for MyHC-slow [25].

MyHC gene activation during embryonic myogenesis is accompanied by parallel upregulation of MLCs and other contractile protein genes. In situ hybridization studies showed that the transcripts for MLC-1emb (*MYL4*) are expressed together with MLC-1fast (the major splicing product of the *MYL1* gene) beginning in the early developmental stages in the mouse embryonic skeletal muscle; their relative levels are similar at E12.5 but MLC-1fast becomes predominant at E15.5 [16]. MLC-2fast (*MYL3*) transcripts are also present early in mouse embryogenesis, with variable temporal and spatial patterns of expression in different muscle groups [26]. In contrast, transcripts for MLC-1slow/ventricular (*MYL4*) and MLC-3fast (another splicing product of the *MYL1* gene) are not detectable in the developing muscles before E15 [16].

### Embryonic/neonatal-to-adult myosin switch

Developmental myosins disappear in most skeletal muscles during the early postnatal development concomitantly with the upregulation of adult fast myosins. In the rat leg skeletal muscles, the transcripts for adult fast MyHCs (MyHC-2A, MyHC-2X, and MyHC-2B, coded by *MYH2*, *MYH1*, and *MYH4*, respectively) are first detected few days after birth by in situ hybridization and become predominant during the subsequent weeks [27]. This switch occurs earlier in the mouse skeletal muscles, as small amounts of adult fast myosin transcripts can be detected even before birth using sensitive RNAase protection assays and by in situ hybridization [28]. However, at the protein level, the fast newborn mouse muscles contain essentially MyHC-neo (about 70 %) and MyHC-emb (about 30 %) with traces of MyHC-slow, as determined by high-resolution gel electrophoresis [29]. The timing of embryonic and neonatal myosin downregulation and adult fast myosin upregulation shows significant variation among body muscles, both at the mRNA [28] and protein level [29]. The elimination of developmental myosin may also vary within the same muscle, for example, neonatal myosin was found to persist longer in type 2A fibers during postnatal development [30]. Interestingly, the timing of downregulation of developmental MyHC isoforms was essentially unchanged in *MYH4* (2B) and *MYH1* (2X) null mice [31].

The switch from developmental to adult fast MyHCs seen in rodent fast muscles takes place also in cultured muscle cells. It has been reported that C2C12 muscle cells, when induced to differentiate upon transfer to low serum medium, first express MyHC-emb, MyHC-neo, and MyHC-slow transcripts, starting at day 1 and peaking at day 2–4 then decreasing, whereas MyHC-2A, MyHC-2X, and MyHC-2B transcripts start to increase at day 2–4 and peak by day 8 (the last time point examined) [32]. However, there are controversial results about the MyHC expression pattern in satellite cell cultures from different skeletal muscles (see [33, 34]), and masticatory-specific myosin heavy chain (Myh16) was detected in cultures of cat jaw muscle but not limb muscle, suggesting that muscle cells from jaw-closing muscles are preprogrammed to express these isoforms during myogenesis in vitro [35].

The developmental switch from developmental to adult MyHCs can be modulated by extrinsic hormonal and neural influences. The embryonic/neonatal-to-adult fast myosin switch is under the control of a thyroid hormone, hyperthyroidism inducing a precocious expression of adult fast myosin heavy chain mRNA and hypothyroidism inducing a delay in this switching [36–38]. In contrast, nerve activity is apparently not necessary for the embryonic/neonatal-to-fast myosin switch [39, 37] but is required to promote the postnatal accumulation of MyHC-slow and the disappearance of MyHC-emb in the slow soleus muscle [19].

The molecular mechanisms controlling the myosin switch during development remain to be established and probably involve specific regulatory sequences associated with the *MYH* gene cluster, where *MYH* genes are arranged in the order: *MYH3*-*MYH2*-*MYH1*-*MYH4*-*MYH8*-*MYH13*. It has been reported that thyroid hormone controls the transition between neonatal and adult fast 2B MyHC by a long non-coding antisense RNA which is transcriptionally regulated during postnatal development and in response to hypothyroidism: this antisense RNA is transcribed from a site within the intergenic region between *MYH8* (MyHC-neo) and the closely associated *MYH4* (MyHC-2B) gene and appears to mediate the transcriptional repression of the *MYH8* gene [40]. A central enhancer located between the *MYH3* and *MYH2* genes has been recently identified [41]. This enhancer, whose function is controlled by six homeoproteins, acts in *cis* by upregulating the expression of fast *MYH* genes (*MYH2*, *MYH1*, and *MYH4*), located downstream of the enhancer, and in *trans* via a long intergenic non-coding RNA (*linc-Myh*) to suppress the expression of *MYH7* (MyHC-slow) [41]. However, it is not known whether this enhancer is also involved in the regulation of developmental myosin genes, *MYH3* and *MYH8*, thus behaving like a *MYH* locus control region (LCR) similar to that present in the *β-globin* locus, or whether other LCRs, associated to the *MYH* gene cluster, control the developmental *MYH* switch.

### Myosin changes in the developing human skeletal muscle

**Fig. 1:**
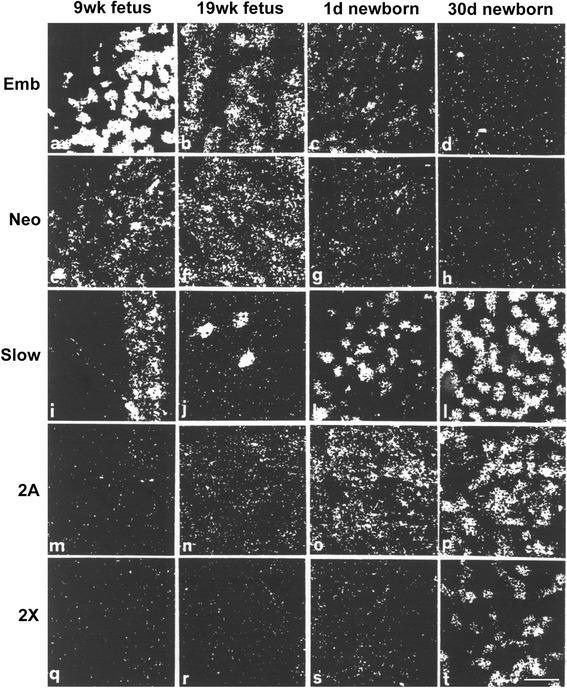
MyHC transcripts in developing human skeletal muscle. The transcripts were revealed by in situ hybridization using probes specific for the following *MYH* genes: *MYH3* (*Emb*, **a**–**d**), *MYH8* (*Neo*, **e**–**h**), *MYH7* (*Slow*, **i**–**l**), *MYH2* (*2A*, **m**–**p**), and *MYH1* (*2X*, **q**–**t**). Muscles examined were quadriceps femoris from 9 and 19-week-old fetuses and vastus lateralis from 1-day- (P1) and 1-month-old (P30) newborns. *Bar* = 30 μm (from [48])

The developmental pattern of myosin isoform expression in the human embryonic and fetal skeletal muscle has been comparatively less investigated. At week 8 of gestation, primary generation fibers with central nuclei are present in the human skeletal muscle, whereas secondary generation fibers are formed after week 10 and become the predominant fiber population by week 21 [42]. MyHC-emb, MyHC-slow, and MyHC-neo transcripts are detectable in the developing skeletal muscle at week 9 (Fig. 1). At the protein level, all primary myofibers express MyHC-emb and MyHC-slow [43, 44], with MyHC-emb being detectable before MyHC-slow in the initial myotubes [45]. The proportion of fibers staining for MyHC-slow decreases from 75 % at week 10 to 3 % at week 21 of gestation, due to the dramatic increase in secondary fibers that initially do not contain MyHC-slow [45]. Secondary generation fibers express only MyHC-emb at week 12, MyHC-neo protein being detected at later stages [45]. Quantitative RNA analysis indicates that *MYH3* transcripts account for about 81 % of all *MYH* transcripts in the human fetal skeletal muscle at week 15 of gestation [46]. At week 16 to 17, a tertiary fiber population has been identified, initially composed of very small myofibers stained by an anti-myosin antibody reactive with adult fast but not with neonatal MyHC [44, 47]. In situ hybridization indicates that MyHC-2A transcripts are weakly expressed at week 19 and more strongly at birth, whereas MyHC-2X transcripts are barely present at birth and are clearly expressed at 30 days after birth (Fig. 1). After week 27, a proportion of secondary fibers starts to express MyHC-slow, and by week 30, about 50 % of all muscle fibers express MyHC-slow, like in adult muscle [45, 44]. In the developing human muscles, both developmental MyHC isoforms are downregulated toward the end of gestation, the corresponding MyHC transcripts are expressed at low levels at birth, and in a 1-month-old infant, MyHC-neo persists only in a few fibers [48] (Fig. 1). In conclusion, most human skeletal muscle fibers, probably more than 95 %, appear to derive from secondary and tertiary waves of myogenesis and their diversification into the fast type 2A or slow type 1 lineage occurs before birth, during the third trimester of gestation, whereas the differentiation of type 2X fibers takes place in the first week after birth.

In the developing human quadriceps, three MLC proteins can be detected by 2D gel electrophoresis between week 7 and 12 [49]. MLC-3fast becomes clearly visible at week 25, when MLC-1emb starts to decrease rapidly. The major change during the third trimester of gestation is the progressive accumulation of the slow isoforms of MLC, so that at birth, the MLC profile is similar to that of adult muscle [49]. MLC-1sa transcripts are also detectable in human skeletal muscles at week 24, though at significantly lower levels compared to adult muscle [50].

### Developmental myosins in adult skeletal muscle

**Fig. 2:**
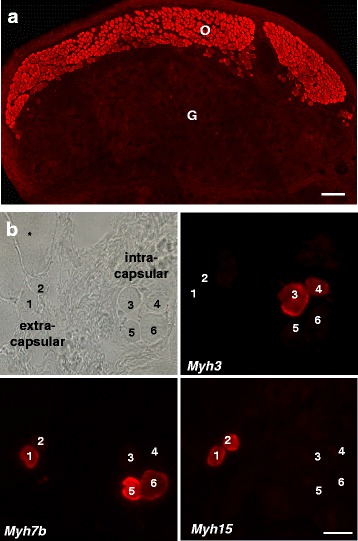
Embryonic MyHC in adult skeletal muscles. **a** Transverse sections of rat extraocular muscle (rectus superior) reacted with a monoclonal antibody specific for MyHC-emb (BF-G6, see [76]). Note that embryonic myosin is expressed in most fibers of the orbital layer (*O*) but only in rare fibers of the global layer (*G*) of the muscle. *Bar* = 100 μm. **b** Embryonic myosin in intrafusal fibers of muscle spindles. Serial sections of rat soleus muscle viewed in phase contrast or stained for MyHC-emb (*MYH3*), MyHC-slow-tonic (*MYH7b*), or MYH15 (*MYH15*). Embryonic myosin is detected in the nuclear chain fibers of a muscle spindle (*3* and *4*) but not in the nuclear bag fibers (*5* and *6*), nor in the extracapsular region of an adjacent spindle (fibers *1* and *2*). Extrafusal muscle fibers (*asterisk*) are unstained. *Bar* = 20 μm (modified from [22])

Developmental MyHCs persist throughout adult stages in a number of fibers present in specialized muscles, including the extraocular muscles [51, 52] and muscle spindles [53, 54, 23], as well as the jaw-closing muscles [55–57] and laryngeal muscles [58]. MLC-1emb/atrial is also present in adult human masseter muscle [55] and is the exclusive or predominant essential MLC associated with MyHC-M (*MYH16*) in the jaw-closing muscles of carnivores and other mammalian species [59, 60]. A recent proteomics study of single fibers from the adult mouse skeletal muscle revealed that traces of MyHC-emb are detectable in all adult myofibers, whereas small amounts of MyHC-neo are present in fast muscle fibers [61]. MyHC-emb has also been detected in the adult human skeletal muscle [62]. In the extraocular muscles, the fibers expressing MyHC-emb are specifically localized in the orbital layer (Fig. 2a) and show variations in expression along the length of the fibers, being more abundant in the distal zones and less abundant in the central zone (see [63]). In these muscles, embryonic myosin is usually co-expressed with other myosin types [63], including the newly discovered MYH15 [22]. In the two fiber types present in muscle spindles, the nuclear chain and nuclear bag fibers, MyHC-emb and MyHC-neo are mostly localized in nuclear chain fibers (Fig. 2b). The embryonic and neonatal *MYH* genes can be induced by hypothyroidism in specific adult muscles [64]. Muscle paralysis induced by resection of the nerve or by tetrodotoxin-induced block of nerve conduction also leads to re-expression of developmental myosins, which occurs specifically in type 2A fibers but is generally restricted to short fiber segments [30].

### Re-expression of developmental myosins in regenerating muscle

**Fig. 3:**
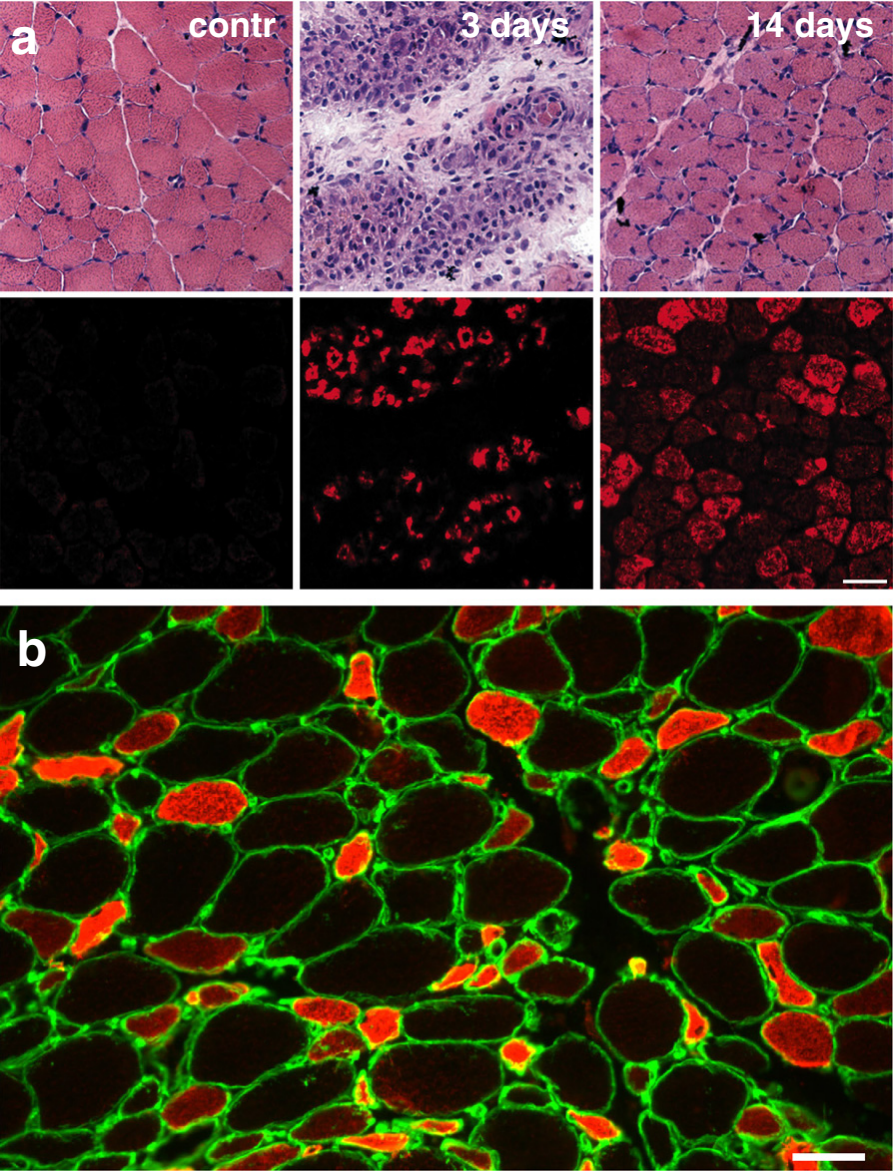
Embryonic MyHC in regenerating muscle fibers. **a** Expression of embryonic myosin in regenerating rat skeletal muscle at various time periods after bupivacaine-induced injury. The progression of muscle regeneration from day 3 to day 14 after injury can be followed in serial sections stained with hematoxylin and eosin (*upper panels*) or immunostained for embryonic myosin (*lower panels*). Note the absence of embryonic myosin in the control muscle. *Bar* = 50 μm (modified from [65]). **b** Regenerating muscle fibers staining for embryonic myosin in human myopathies. Section of human muscle biopsy from a patient with polymyositis stained for MyHC-emb (*red*) and laminin (*green*). Note the large number of regenerating muscle fibers reactive for embryonic myosin. *Bar* = 50 μm (courtesy of Elena Pegoraro)

Skeletal muscles can efficiently regenerate after different types of injury (see [65] for a review). Muscle regeneration is mediated by the satellite cells present under the basal lamina of the muscle fibers, which are activated after injury and undergo proliferation and fusion, thus forming new muscle fibers. Regenerating muscle fibers re-express developmental isoforms of myosin, troponin, and other muscle proteins [66, 67, 3]. Embryonic and neonatal MyHCs are detected in newly formed regenerating myofibers at 2–3 days after injury and persist for 2–3 weeks (Fig. 3a). MLC-1emb is also transiently expressed in regenerating skeletal muscles [68]. Re-expression of developmental myosins can be revealed in a variety of conditions that involve muscle degeneration/regeneration events, including injection of the snake venoms notexin and cardiotoxin [69, 70], chronic denervation [71], or muscle damage induced by chronic electrical stimulation [72]. The presence of developmental myosins thus represents a useful marker of muscle regeneration in animal models of muscle disease, such as the dystrophin-deficient *mdx* mouse model of muscular dystrophy [73] and in human myopathies, such as Duchenne muscular dystrophy [74] or polymyositis (Fig. 3b). The presence of embryonic myosin can also be a useful marker in the diagnosis of rhabdomyosarcoma [75, 76].

The switch from embryonic/neonatal-to-adult fast myosins in regenerating muscle is independent of innervation, whereas the switch to slow myosin is controlled by slow nerve activity [69, 66, 70]. In an extensively used model, with muscle injury induced by bupivacaine injection in the rat slow soleus muscle, regenerating myofibers express only embryonic and neonatal myosin transcripts at day 2–3 after injury but, at day 4, start to express adult fast myosin mRNAs. However, in the presence of the nerve, the slow myosin is rapidly upregulated and fast myosin transcripts are downregulated, whereas in the absence of the nerve, adult fast myosins continue to accumulate and slow myosin transcripts remain undetectable [77]. This process is mediated by the pattern of nerve activity, as it can be reproduced by electrical stimulation of regenerating muscle using a stimulation pattern that mimics the endogenous slow motor neuron activity [78]. However, regenerating fast and slow muscles respond differently to the same stimulation pattern, supporting the possibility that the embryonic/neonatal-to-adult fast or slow myosin switch reflects the existence of intrinsic differences between satellite cell populations in the various fiber types. This interpretation is consistent with a number of studies on cultured muscle cells; however, this issue is outside the scope of this review.

### Human congenital disorders due to mutations of embryonic and neonatal myosins

The crucial role of embryonic and neonatal myosin during human development has more recently been demonstrated by the pathological consequences of *MYH3* and *MYH8* mutations (see [79]). Mutations in the *MYH3* (MyHC-emb) gene are responsible for some types of distal arthrogryposis (DA) syndromes, congenital disorders characterized by multiple limb contractures [80]. *MYH3* gene mutations have been associated with two major DA syndromes, DA2A and DA2B/DA1. Freeman-Sheldon syndrome (FSS, DA2A) is characterized by facial contractures and congenital scoliosis, in addition to contractures of the limbs. This is the most severe of the DA syndromes and patients require nutritional, surgical, and rehabilitative intervention [81]. FSS was also known as the “whistling-face syndrome”, because the lips appear pursed or pinched leaving only a small oral opening. In fact, to date, the only identified cause of FSS is mutation in the MYH3 gene. DA2B (Sheldon-Hall syndrome, SHS) and DA1, which appear to represent the extremes of the same phenotypically variable and genetically heterogeneous condition, can also be due to *MYH3* mutations [82]. However, DA2B and DA1 can also be caused by mutations in *TNNI2*, coding for fast troponin I, *TNNT3*, coding for fast troponin T, and *TPM2*, coding for β-tropomyosin.

**Fig. 4:**
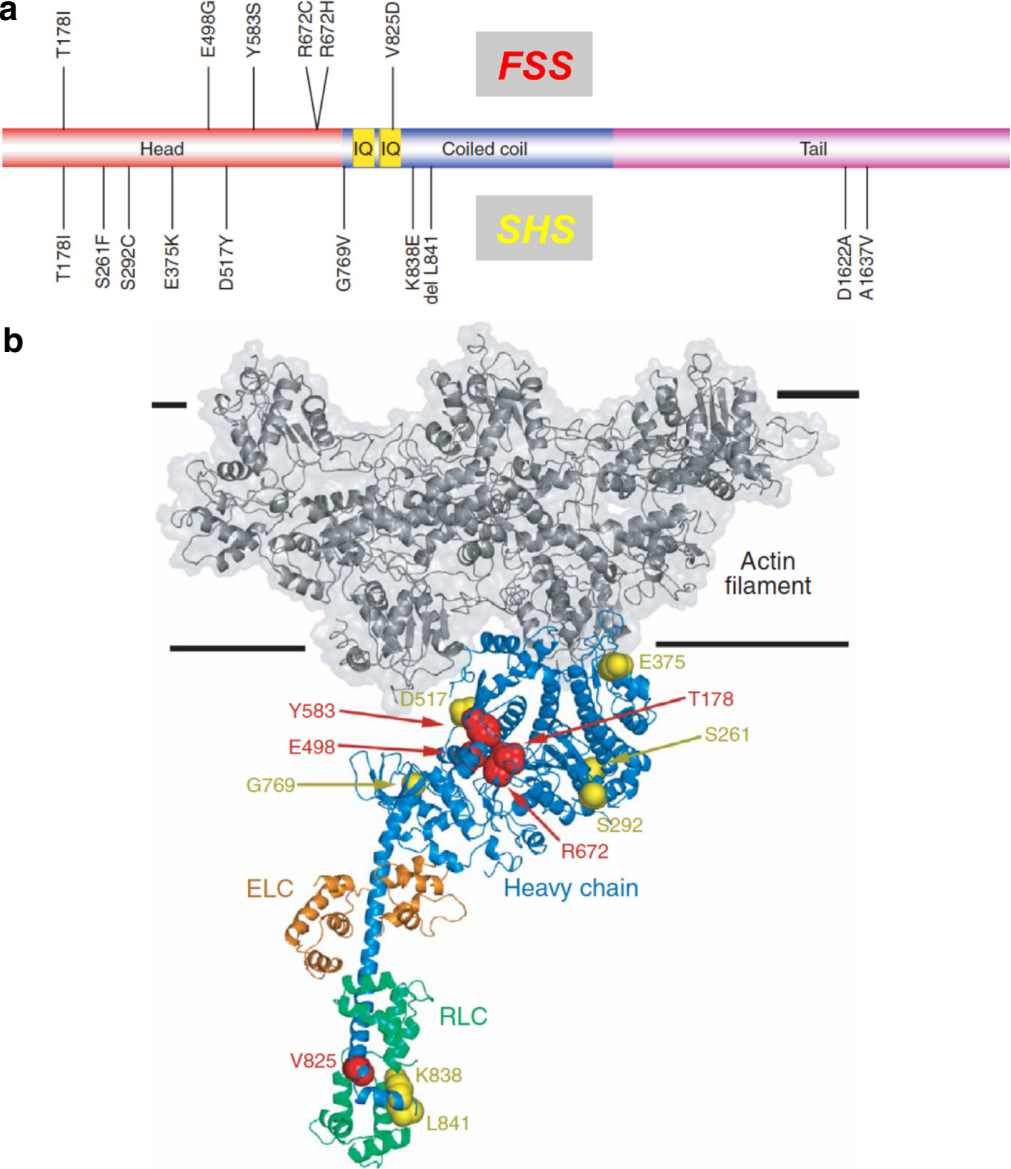
*MYH3* mutations causing distal arthrogryposis. **a** Scheme of the embryonic myosin molecule showing the sites of different mutations causing Freeman-Sheldon syndrome (*FSS*, *above*) and Sheldon-Hall syndrome (*SHS*, *below*). Note that most mutations localize to the head domain of the myosin molecule, and that mutations causing FSS differ from those causing SHS. **b** A model of the actin-myosin complex. A portion of the actin filament comprising five actin monomers is shown as a dark gray ribbon. Myosin heavy chain (*Heavy chain*), essential light chain (*ELC*), and regulatory light chain (*RLC*) are shown as *blue*, *orange*, and *green ribbons*, respectively. *MYH3* mutations causing distal arthrogryposis are shown with oversized space-filling atoms, with FSS mutations colored *red* and SHS mutations *yellow* (modified from [80])

Most *MYH3* mutations in DA2A and DA2B do not overlap, suggesting that there is a relationship between *MYH3* genotype and phenotype (Fig. 4), and also within DA2A several aspects of the phenotype are associated with specific mutations [80]. Three *MYH3* mutations involving conserved residues, T178I, R672H, and R672C, account for more than 90 % of the *MYH3* mutations that cause FSS, with T178I being the most severe and R672C the least [81]. However, T178I has also been associated with SHS. Both R672 and T178 residues map to a groove adjacent to the nucleotide binding site, suggesting that mutation of these residues may alter the active site surrounding the nucleotide binding site. In contrast, residues mutated in SHS generally localize to surfaces that may interact with other proteins of the contractile apparatus such as actin and troponin: this could explain why a similar SHS phenotype can be caused by *TNNI2* and *TNNT3* mutations (Fig. 4).

*MYH8* (MyHC-neo) mutations are responsible for another form of distal arthrogryposis (DA7), referred to as the trismus-pseudocamptodactyly syndrome (TPS) because the patients cannot open the mouth fully (trismus) and show an unusual camptodactyly (flexion of the fingers) that is evident only on dorsiflexion of the wrist (i.e., pseudocamptodactyly). In contrast to the large number of *MYH3* mutations causing FSS and SHS, only a single *MYH8* mutation (R674Q) has been identified in different families with trismus-pseudocamptodactyly syndrome [83, 84]. The affected residue R674, which because of different numbering corresponds to R672 in the MYH3 gene described above, is conserved in different vertebrate species and in different *MYH* gene coding for sarcomeric myosins. This residue is localized near the ATP binding site and may thus interfere with myosin catalytic activity. In the family described by Veugelers et al. [84], TPS was found to be associated with manifestations typical of the Carney complex, including the presence of cardiac myxomas, suggesting a possible role of the *MYH8* gene in cardiac development. However, this association was not found in the families reported by Toydemir et al. [83], and TPS was never observed in large collections of Carney complex cases [85].

How can one explain the congenital contractures induced by mutations in developmental myosins? A plausible interpretation is that *MYH3* or *MYH8* gene mutations interfere with myosin’s catalytic activity due to the dominant negative effect of the mutated allele, thus causing defects in myofiber force production in utero. Active movements of the embryo are required for the normal development of the joints, as shown by classic studies in the chick embryo [86, 87]. These studies showed that muscle paralysis induced in ovo by neuromuscular blocking agents, such as curare or botulinum toxin, causes arthrogryposis. The orofacial dysmorphisms induced by *MYH3* or *MYH8* gene mutations might reflect a similar role of the contraction of facial expression muscles in shaping the form of the face during fetal development.

The view that mutations of *MYH3* and *MYH8* lead to hypocontractility of fetal muscles has received support by two recent findings. First, the alteration of the cross-bridge turnover in patients carrying R672C mutation has been confirmed by a detailed analysis of myofibril and single-fiber mechanics [62]. Second, preliminary results with isolated myosin S1 (subfragment 1), the portion of the myosin molecule comprising the myosin head and lever arm, which is sufficient to drive actin sliding movement in in vitro motility assays, have shown that several kinetic parameters of the cross-bridge cycle are altered in the presence of R672C, R672H, and T178I FSS-causing mutations [88].

### Contractile properties of embryonic and neonatal myosins

**Fig. 5:**
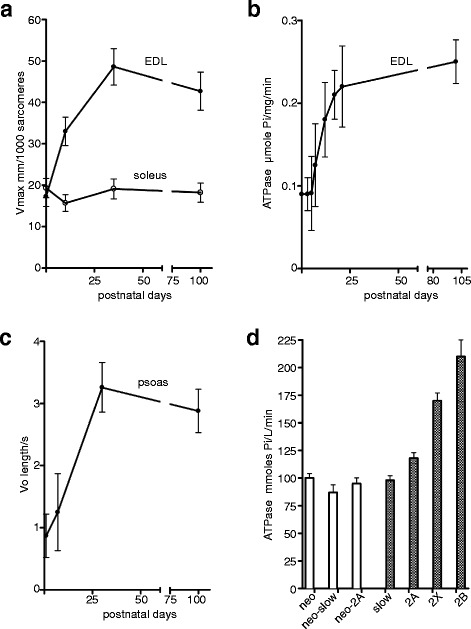
Kinetic properties of neonatal myosin. **a**, **b** Postnatal changes in maximum shortening velocity (**a**) and ATPase activity (**b**) in rat extensor digitorum longus (*EDL*) muscle. Note that the developmental replacement of neonatal myosin with adult fast myosin during the first week after birth is accompanied by a twofold increase in V_max_ and ATPase activity. (Panel **a** redrawn from Table 1 of [91], panel **b** redrawn from Figure 3 (a) of [92]). **c** Unloaded shortening velocity of single fibers of rabbit psoas increases during postnatal stages concomitantly with the replacement of neonatal with adult fast isoforms (redrawn from data in text and in Figure 2 of [96]). **d** Myofibrillar ATPase activity of single fibers isolated from neonatal and adult rat diaphragm muscle and identified in relation to their MyHC isoform composition. Note the lower ATPase activity of neonatal compared to adult fast fibers (redrawn from data in Figure 4 of [98])

Pioneering studies in the 1960s showed that a transition in contractile properties occurs around or just after birth in cat [89, 90] and rat muscles ([91, 92], see [93] for a review), as depicted in Fig. 5a, b. During the first week of postnatal development, isometric force increases in both slow and fast muscles, while maximum shortening velocity increases in fast but not in slow muscles. The increase in strength could be explained by addition of myofibrils in parallel (but see below); however, the change in maximum shortening velocity points to changes in myosin isoforms, which are assumed to be the major determinants of maximum shortening velocity and ATPase activity [94]. Further support to this interpretation was given by the observation of a parallel increase in myosin ATPase activity during development [92], whereas the acceleration in rate of tension rise and the reduction of twitch time parameters presumably reflect a convergent contribution of changes in myosin kinetics and maturation of sarcoplasmic reticulum [92, 95].

The changes due to myosin isoform developmental replacement were studied in rabbit psoas single fibers [96]. At birth, neonatal myosin is predominant and is progressively replaced by adult fast isoforms. The link between myosin isoform replacement and changes in contractile properties, maximum shortening velocity, and ATPase activity has been analyzed in single muscle fibers where MyHC expression was determined by gel electrophoresis. In rabbit psoas, replacement of neonatal MyHC with adult fast, mainly 2X, isoforms is associated with a threefold increase in maximum shortening velocity [96] (see Fig. 5c). In the rat diaphragm, neonatal myosin is the predominant isoform in the first two weeks after birth, although rarely expressed alone in individual fibers, but more often associated with fast 2A myosin [97–99]. Fibers expressing predominantly neonatal myosin show values of shortening velocity and ATPase activity comparable to slow fibers and much lower than fast 2X and 2B fibers (Fig. 5d). The disappearance of neonatal myosin is associated with an increase in ATP consumption rate [98] and increase in power output [99].

The view that, in mammals, neonatal myosin has kinetics similar to 2A myosin but slower than 2X and 2B myosins has received support from the experiments on recombinant human myosin S1 motor domain expressed in C2C12 myotubes [100]. An additional interesting feature emerging from Resnicow et al. [100] data is that *K*_*m*_ values for actin are much greater for developmental than for adult myosins. This suggests even lower values of ATP hydrolysis rate for immature myofibrils in conditions other than maximal actin activation.

The functional features of embryonic myosin are still little known. The study by Resnicow et al., however, shows that the kinetics of embryonic myosin are slower than those of neonatal myosin, both for ATPase rate and for actin filament sliding velocity [100]. Although the interpretation of this result is complicated by the finding that embryonic myosin motor domain did not bind any light chain when expressed in C2C12 myogenic cells, an independent study points to the same conclusion [46]. Purified myosin and intact myofibrils were prepared from human muscle samples obtained from four fetuses of the age of 12–15 weeks post conception. Quantitative PCR and protein analysis showed that MyHC-emb was largely predominant, above 80 % of the total myosin present. Compared to psoas rabbit myosin (likely a mixture of 2X and 2B myosins), the actin filament velocity of the human embryonic myosin was more than three times lower. Taking into account that rabbit myosins are approximately two times as fast as human embryonic muscle myosin(s) [101], one can assume that gliding speed of actin filaments on human embryonic myosin is at least 1.5 times lower than on fast human myosin.

Intact myofibrils also allowed determination of force and rate of force development and decline. Force developed by myofibrils containing embryonic myosin was found to be more than ten times lower than that developed by adult human myofibrils [46]. No data are available for the ability to develop force of myofibril containing neonatal myosin, but the results obtained on embryonic myosin suggest that the increase of active force during development may be due not only to accumulation of myofibrils in parallel but also to the transition of myosin isoforms.

While the kinetic properties of the contractile response are almost exclusively linked to the MyHC isoforms, the force development might also be significantly affected by other proteins present in the myofibrillar apparatus. Developmental changes in MLC gene expression (see above) may be relevant. In the thin filament, embryonic and neonatal skeletal muscle may also express unique isoforms: for example, cardiac troponin T (TnT) is expressed in embryonic skeletal muscle and unique TnT isoforms, presumably derived by alternative splicing of the fast skeletal muscle TnT gene, are detected in fetal and neonatal muscle [67].

### Functional significance of developmental myosins

One relevant question remains unanswered: what is the advantage (or necessity), if any, of having specific myosin isoforms during muscle development. Two distinct interpretations can be considered. One possibility is that developmental myosins have structural characteristics appropriate for myofibril formation during myogenesis, both in the embryo and during muscle regeneration in the adult. According to the premyofibril model of myofibrillogenesis developed by Sanger from studies in avian cardiac and skeletal muscle cells and recently confirmed in mouse skeletal muscle cells [102], myofibril assembly in premyofibrils is characterized by bands of class II non-muscle myosin alternating along actin fibers with bands of muscle-specific α-actinin. The transition from the premyofibril to the nascent myofibril is marked by the addition of class II muscle (sarcomeric) myosin, but it is not known whether the presence at this stage of MyHC-emb is an obligatory step for myofibrillogenesis to occur in skeletal muscle cells. Knockout experiments in vivo or knockdown experiments in cultured muscle cells would be required to address this question. It should be stressed that myofibrils essentially identical to those present in skeletal muscle are formed in the absence of MyHC-emb in developing cardiac muscle cells, which contain only MyHC-β/slow and MyHC-α [103]. Knockout or knockdown experiments could also be used to determine whether embryonic and neonatal isoforms have redundant functions, so that one of them is able to fully compensate for the lack of the other.

Another possibility is that embryonic and neonatal myosins have unique properties adapted to the prenatal developmental environment. For example, it is well known that fetal hemoglobin has a greater oxygen affinity than adult hemoglobin due to specific embryonic and fetal globins, whose presence contributes to transplacental oxygen flux in the context of a relative hypoxic intrauterine environment [104]. Developmental switching of contractile proteins might also be affected by oxygen tension. In cardiac muscle, hypoxia was shown to reactivate gene expression programs of early cardiac development, with upregulation of MyHC-slow (*MYH7*) and downregulation of MyHC-α cardiac (*MYH6*), both in ventricles from rats exposed to hypobaric hypoxia and in neonatal rat cardiomyocytes incubated in a hypoxic chamber [105]. In cultured skeletal muscle cells, hypoxia was found to stimulate the expression of MyHC-slow via HIF-1α [106]. The developmental switching of troponin I from the slow skeletal to the cardiac isoform, that is known to modulate the calcium sensitivity of the contractile apparatus, has been associated with the greater resistance to hypoxia and acidosis of the fetal and neonatal heart (see [107]). However, to our knowledge, there is no comparative study on the effect of hypoxia and acidosis on the function of developmental and adult myosins in skeletal muscle. The low ATPase rate typical of neonatal, and even more, embryonic myosin might suggest that these myosin isoforms allow a contractile activity at a very low energetic cost.

An alternative possibility is that load-bearing properties of developmental myosins play an important role in the transitions of myosin during development, as fetal muscles contract against a very low load compared to postnatal muscles [108]. It is tempting to speculate that fetal tendons, joints, and bones require the mechanical stimuli produced by muscle contraction for their correct growth but, at the same time, cannot bear excessive strains, and embryonic myosin might have appropriate properties in this respect. Accordingly, it has been speculated that the persistence of developmental myosins in the extraocular muscles may be related to the fact that oculorotatory muscles contract against a much lower load compared to other skeletal muscles [51]. This interpretation could be tested by specific experimental approaches. In particular, it will be crucial to determine the contractility of embryonic and neonatal myosin by loaded in vitro motility assays and single-molecule analyses with a dual-beam laser trap (see [109]).

## Conclusions

The presence of unique MyHCs and MLCs in developing skeletal muscle and their re-expression in regenerating muscle was first reported in the late 1970s to early 1980s. During the subsequent years, the gene coding for embryonic and neonatal myosins were characterized, their expression patterns were defined, and the factors involved in the developmental-to-adult myosin switch were identified. However, the physiological significance of developmental myosins remained completely unclear until 2006, when embryonic (*MYH3*) mutations were first reported to cause specific syndromes characterized by congenital joint contractures. This finding has opened up a new phase of research aimed at dissecting the functional properties of embryonic and neonatal myosins and the consequences of their mutations. Analyses on myofibrils and single fibers, and especially on isolated myosin S1, are expected to define the kinetic parameters of the cross-bridge cycle of developmental myosins and their response to loading conditions, thus addressing the unsettled question of why specific myosin isoforms are needed during muscle development.

## Abbreviations

DA: distal arthrogryposis
FSS: Freeman-Sheldon syndrome
MLC: myosin light chain (protein)
*MYH*: myosin heavy chain (gene)
MyHC: myosin heavy chain (protein)
*MYL*: myosin light chain (gene)
SHS: Sheldon-Hall syndrome
TPS: trismus-pseudocamptodactyly syndrome
